# m-SFT: A Novel Mobile Health System to Assess the Elderly Physical Condition

**DOI:** 10.3390/s20051462

**Published:** 2020-03-06

**Authors:** Raquel Ureña, Francisco Chiclana, Alvaro Gonzalez-Alvarez, Enrique Herrera-Viedma, Jose A. Moral-Munoz

**Affiliations:** 1 Institute of Artificial Intelligence (IAI), School of Computer Science and Informatics, De Montfort University, Leicester LE1 9BH, UK; chiclana@dmu.ac.uk; 2 Andalusian Research Institute on Data Science and Computational Intelligence (DaSCI), University of Granada, 18071 Granada, Spain; viedma@decsai.ugr.es; 3 Department of Computer Science, University of Cadiz, 11519 Puerto Real, Spain; alvaro.gonzalezalvarez@alum.uca.es; 4 Department of Nursing and Physiotherapy, Universidad de Cádiz, 11009 Cádiz, Spain; joseantonio.moral@uca.es; 5 Institute of Research and Innovation in Biomedical Sciences of the Province of Cádiz (INiBICA), Universidad de Cádiz, 11009 Cádiz, Spain

**Keywords:** senior fitness test, physical condition, elderly, physical condition assessment, m-health, mobile health, healthy aging

## Abstract

The development of innovative solutions that allow the aging population to remain healthier and independent longer is essential to alleviate the burden that this increasing segment of the population supposes for the long term sustainability of the public health systems. It has been claimed that promoting physical activity could prevent functional decline. However, given the vulnerability of this population, the activity prescription requires to be tailored to the individual’s physical condition. We propose mobile Senior Fitness Test (m-SFT), a novel m-health system, that allows the health practitioner to determine the elderly physical condition by implementing a smartphone-based version of the senior fitness test (SFT). The technical reliability of m-SFT has been tested by carrying out a comparative study in seven volunteers (53–61 years) between the original SFT and the proposed m-health system obtaining high agreement (intra-class correlation coefficient (ICC) between 0.93 and 0.99). The system usability has been evaluated by 34 independent health experts (mean = 36.64 years; standard deviation = 6.26 years) by means of the System Usability Scale (SUS) obtaining an average SUS score of 84.4 out of 100. Both results point out that m-SFT is a reliable and easy to use m-health system for the evaluation of the elderly physical condition, also useful in intervention programs to follow-up the patient’s evolution.

## 1. Introduction

The aging of the population is a global phenomenon that presents its highest impact in the developed countries. In the case of the European Union, the average life expectancy is over the 80 years, an increase of ten years since 1970, with the senior population exceeding 80 years old being the fastest growing segment. In fact, this segment is expected to represent 20% of the older population by 2050. Besides, both the aging of the population and the decreasing birth rates are motivating that the demographic old-age dependency ratio, (the ratio between the people aged 65 or above with respect to those aged 15–64), to be expected to exponentially increase in the upcoming decades, from about 2% in 2010, it rose to 29.6% in 2016 and is projected to eventually reach 51.2% by 2070. Consequently, in the case of the EU, it would evolve from four working-age people for every person aged over 65 years in 2010 to around two working-age persons over the projection horizon [1,2].

This change in the age profile implies a population with more physical limitations. This fact entails an increasing burden for the governments, making long term sustainability of the pubic health system a key challenge for the countries’ economies [3]. For example, when an individual from a young segment of the population becomes aware of a illness, she receives a diagnosis and usually a treatment converts the condition into a short-term illness [4]. Conversely, in the case of the elderly, 92% have at least one chronic disease, and 77% suffer from at least of two [5].

Therefore, there is an imperative need to develop innovative solutions that enable the aging generation continue independent longer and to make them responsible of their own health habits and physical function. That implies a shift, from treatment towards prevention, in the public health care paradigm with regards to the age-related diseases [6].

With this regard, it has been demonstrated that physical activity, defined in [7] as “any bodily movement produced by skeletal muscles that results in energy expenditure above the basal resting level”, presents high benefits for the people at both physiological and psychological levels [8], including the elderly. In the case of the elderly, the regular practice of physical activity prevents functional decline, osteoporosis, frailty, falls, and fractures, decreases the risk of cardiovascular disease, type 2 diabetes, and certain cancers, and reduce the risk of premature mortality. However, in spite these undoubted benefits, this segment of the population remains largely inactive. Thus, finding effective ways to increase and maintain physical activity levels in older people over prolonged periods constitute a challenge [9]. With this particular, technologies that enable ongoing exercise are gaining importance as the proportion of older people in the population increases making the resources to provide rehabilitation care to become scarce [10]. However, several studies support the fact that in order to obtain the maximum benefits of the training, the technology prescription and health coaching support requires to be tailored to the functional and personal characteristics of each individual [11]. In consequence, developing tools that asses the physical condition in an easy, and cost-effective way [8], becomes more than necessary.

This is a context where the use of e-health plays a key role. According to [12] “E-health is an emerging field in the intersection of medical informatics, public health and business, referring to health services and information delivered or enhanced through the Internet and related technologies”. Furthermore, according to the World Health Organization (WHO), “the strengthening of health systems through e-Health reinforces fundamental human rights by improving equity, solidarity, quality of life and quality of care.” A particular application of e-health is the case of m-health, that is, the use of smartphone applications as tools to deliver medical information and provide care. m-health is becoming a feasible and helpful utility not only for perioperative patient care but also for patient evaluation and evolution assessment [13].

In this contribution we leverage the power of m-health to develop a mobile Senior Fitness Test (m-SFT), a new m-health system to assess the elderly physical condition. m-SFT consists of an electronic implementation of the Senior Fitness Test (SFT) proposed by Rikli et al. in [14]. To do so in a cost-effective and easy-to-use way, the proposed approach uses the built-in sensors in an inexpensive android smartphone [15] to automatically evaluate, record and control the individual’s muscle strength, the lower and upper limbs flexibility, the aerobic endurance, and the agility. In that way, this test was recently implemented in a smartphone-based system to monitor the elderly daily physical activity [16].

This contribution is structured as follows: Section 2.1 presents an overview of the m-health apps focusing specially on the elderly. Section 2.2 describes the fundamental principles of SFT, whereas Section 3.1 presents the proposed m-health system describing the sensor set up. An evaluation of both the technical reliability and the usability of the proposed system is shown in Section 3.2. Finally the discussion and conclusions and the future research challenges that this contribution poses are pointed out in Section 4 and Section 5.

## 2. Material and Methods

### 2.1. Related Work

m-Health has been defined by the WHO as “medical and public health practice supported by mobile devices, such as mobile phones, patient monitoring devices, personal digital assistants (PDAs), and other wireless devices” [13]. m-Health includes the utilization of all the smartphone’s core utilities, ranging from voice and short messaging service to localization system, built in movement sensors and external wearable devices, i.e., activity monitoring bracelets and smart watches, connected via bluetooth. From the economical point of view, given the almost omnipresent availability of mobile technologies, the number of m-health apps is increasing exponentially, for example, in 2016 the global number of m-health apps reached 259,000, and the rise is expected to continue. So far, US represents the largest m-health market whereas Asian-Pacific region, Latin America and Europe constitute increasing markets predicted to grow within the next five years. In fact, by 2022, the global market for m-health apps is expected to reach 102.43 billion [17]. From the health perspective, m-Health enables non-stop health monitoring at both individual and population level, and may encourage healthy behaviours that might reduce or even prevent health problems [18]. The actual m-health market can be widely classified in the following groups:

- Chronic care management apps. This category contains the apps designed to deal with Chronic diseases and their symptom. This category enclosed the apps for managing blood pressure, glucose levels for diabetes, mental health and other illnesses. Besides the applications in the public app markets, from the research perspective various interventions have been proposed in the context of clinical studies, such as depression treatment [19], diabetes control [20], hypertension control [21], and psychological support [22,23].
- **Healthcare and Fitness Apps**. This type of applications seeks for a behavioural change in the users, by tracking their habits with respect to the meals and the sport and providing tailored recommendations. They can be classified in two wide groups: (i) Apps that track the calorie intake such us Lifesum [24]. (ii) Apps that register physical activity using not only the sensors in the phone but other weareable devices such us activity bracelets or smartch watchs. Example of this apps are GoogleFit [25] or Endomondo [26] among others. Among the clinical studies we can remark the one in [27] that aims for a reduction of calorie intake by use of personal digital assistant applications for diet and exercise, or the one in [28] which consist on mobile phone application intervention to increase physical activity levels.
- **Medication Management Apps**. Within this group they are comprised the apps that keep track of medication intake in order to improve its adherence among patients, they are specially useful among the elderly.
- **Personal Health Record Apps**. Among this category we can find the applications that allow patients to store their medical conditions data, allergies etc. and share it with their doctors.

An increasing sector of the market consists of apps designed for the elderly that can be found in the main app catalogs, Google Play and Apple store [6]. Most of these apps are designed for this following two main purposes:

- **Emergency situations detection**. This sector involve the apps that are able to detect if the elderly is in a danger situation, i.e., falls or disorientation, if this is the case, the app fire a flag to the elderly’s caregivers or relative or to the emergency services to assist the person in danger.
- **Behaviour changing and physical tele-rehabilitation**. Among this category we can find out as well some e-health platforms that provides advice and training exercises on how to recover from certain problems. For example activehip [29] is a tele-rehabilitation platform designed for elderly patients recovering from a hip fracture. DIGIREHAB, [30] that is a Danish platform that provides objective assessment of each patients’ need for assistance and his/her general level of ability. Together, these assessments form a precise image of the patients’ physical potential for rehabilitation. We can find out as well other prototypes of mobile solutions for the elderly population that suffers from low vision based on a digital image enhancement [23,31,32,33,34].

In the field of the test applications we can point out various solutions that requires of different sensors or smartphones fixed to the body (e.g., the lower back), for example the instrumented versions of the commonly-used clinical test Timed Up-and-Go [35] or a digital version of the trust endurance presented in [36]. Furthermore, a web based implementation of the aforementioned Senior Fitness Test [14]. that can be used for entering and analyzing the SFT test scores, creating individual or aggregated reports, and generating program outcome statistics has been proposed in [37].

In the light of the existing m-health apps we can conclude that the majority of m-health applications for the elderly are done either with reminding purposes either with advice purposes, but there is no application that carries out the assessment of the physical condition of the elderly in an automatic way using uniquely an inexpensive android mobile phone.

### 2.2. Senior Fitness Test

The Senior Fitness Test consists of a battery of test items that covers up the major components of fitness for older adults. In concrete, it evaluates the physical attributes that are required to perform daily activities in later life in terms of strength, endurance, flexibility, agility, and balance [14]. The main advantages of SFT are based on the facts that it is easy to understand, quick to administer, and safe. Furthermore, in comparison with other tests, it requires a lower number of tools to be performed.

The SFT is composed of eight test item in order to asses the muscle strength, the lower and upper limb flexibility, the aerobic endurance, and the agility. In Table 1, the different test items that composed the SFT are described in detail; furthermore, the values reported for Rikli and Jones’s [14] sample are stated.

## 3. Results

### 3.1. The Proposed App, m-SFT

This contribution proposes a new m-health platform to automatically asses the elderly physical condition by means of the built in sensors in an Android phone. The choice of this Operating System to develop our proposal is based in both its bigger implantation in the market (android smart-phones constitute the 92% of the mobile phones in the Spanish market) and the availability of low cost devices. In the following the technical details on how this app can be implemented are explained.

#### 3.1.1. App Implementation

**Figure 1:**
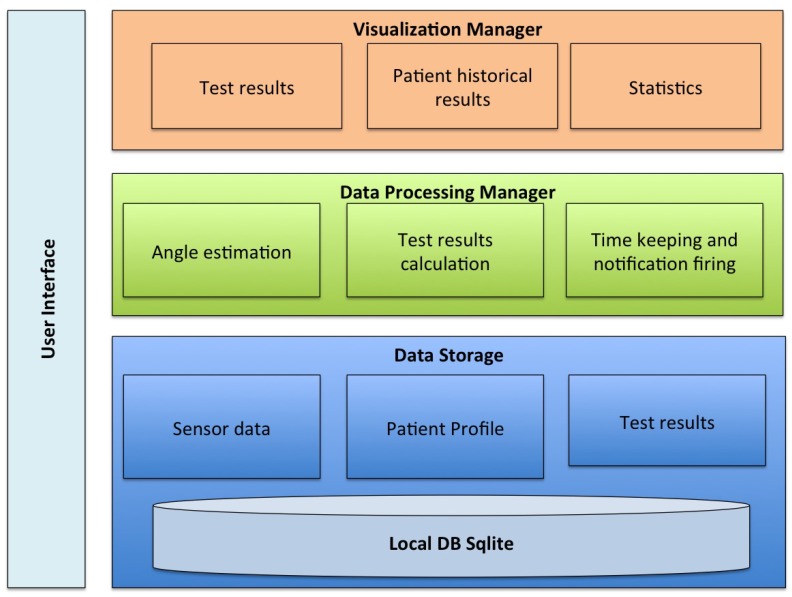
m-SFT architecture.

The architecture of the proposed system is composed of four interconnected layers, as depicted in Figure 1, namely the data storage manager, the data processing manager, visualization manager and the user interface, UI.

**Figure 2:**
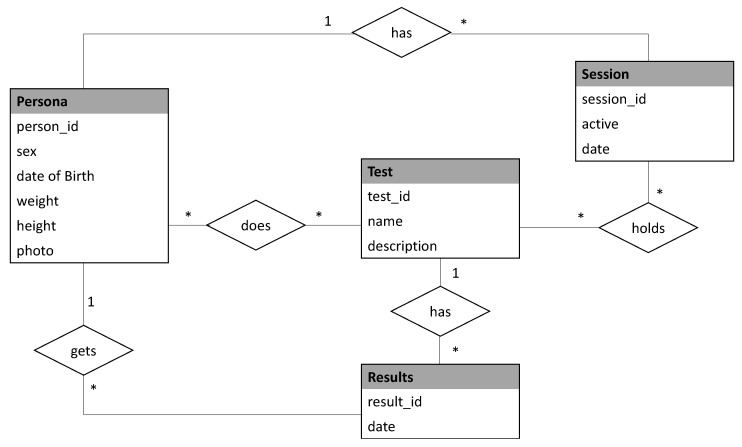
Diagram–entity relationship for the database.

The data storage manager implements a local SQLite database [38] stored on the mobile phone built in memory. This way, the fully off line working capability is ensured. This database keeps one register for each patient storing his/her ID, age, gender, contact information, and the test results. The entity-relationship diagram for the database is depicted in Figure 2. The data base is composed of fourtables namely Persona, Session, Test, and Results. The table Persona stores the user profile information. The table Session includes the information related with each of the test sessions that a given user accomplishes. The table Test contains the type of test and its description and finally the table Result stores the results of each test item for each session for each user. Moreover the information coming from the sensors during the execution of the test, detailed in the following subsection, is buffered and periodically stored in the sensors’ table, in order to ensure efficiency and to facilitate the computation. Once the test is completed and the results are calculated, these results are updated in the corresponding patient table.

The visualization manager provides graphical representation of the historical test results that are depicted in the UI, as it is shown in Section 3.1. This manager is developed on top of the MPAndroidChart [39], an open source library for statistical graphics.

The data processing manager is in charge of the interface with the sensors, estimating the angles and the repetition for each one of the test elements. In the following subsection we detail how this interface has been implemented. Furthermore in Section 3.1.3 we discuss the app UI.

#### 3.1.2. Sensor Interface

The Android platform provides a range of built in sensors that allow to monitor the device motion. These sensor can be broadly classified into hardware-based and software-based and their availability varies depending on the device model. Among the hardware-based sensor we can find both the accelerometer and the gyroscope, whereas the gravity, linear acceleration, rotation vector, significant motion, step counter, and step detector sensors could be either hardware-based or software-based depending on the device. The majority of the devices always include the accelerometer and some of them the gyroscope. The availability of the software-based sensors depends on the built in hardware-based ones, being the last ones the data source for the former [40]. In the case of m-SFT, in order to recognize the movement and the repetitions for the different test items, the proposed system uses the gravity sensor. This is a software-based sensor that asses the effect of the earth gravity acceleration combining both the output from the accelerometer and the gyroscope to remove the linear acceleration. This sensor’s output consists on a three dimensional vector indicating the direction and magnitude of the gravity in m/s${}^{2}$ in the direction of the three spatial axis, *x*, *y* and *z* [40]. The gravity sensor is available in each Android device with Android 2.3 (API nivel 9). The release of this version of Android date from December 2010. Therefore the majority of the Android Devices manufactured after 2010 includes the gravity sensor [41]. Therefore, the reliability of m-sft is guaranteed for all the devices that has Android Version 2.3 or older.

In the following how the sensor set up is leveraged to monitor each one of the items that composed the Senior Fitness test is detailed.

**Figure 3:**
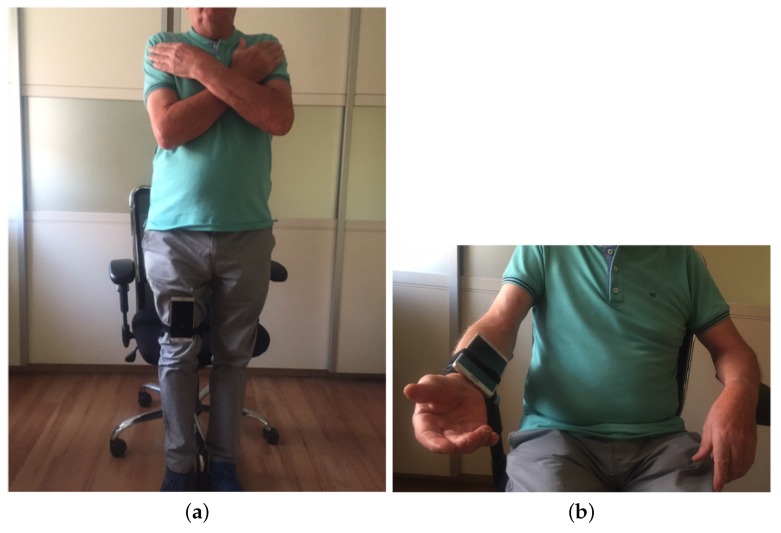
Positions of the mobile phone for the test: (**a**) Leg; (**b**) Arm.

**Figure 4:**
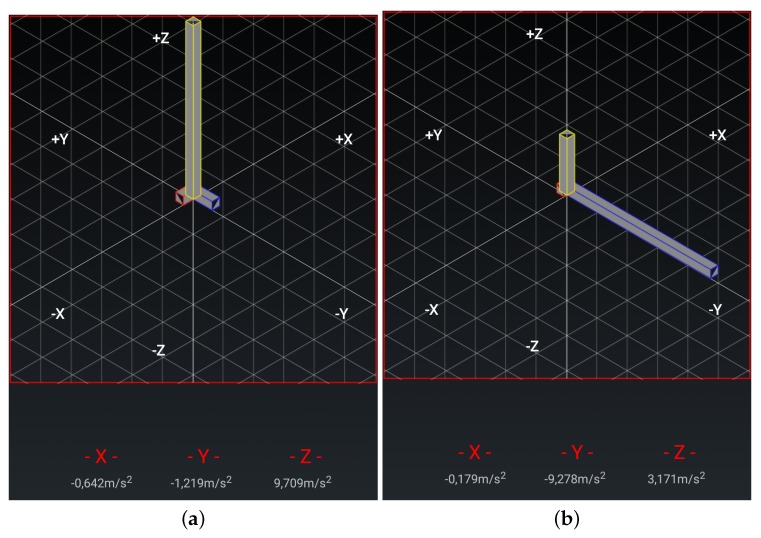
Gravity for the legs strength test: (**a**) User sit; (**b**) User standing.

**Figure 5:**
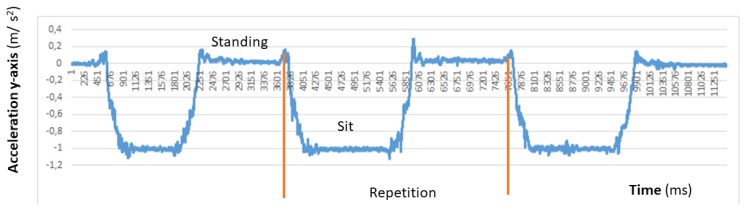
Acceleration in the *Y*-axis direction during the legs strength test.

**Figure 6:**
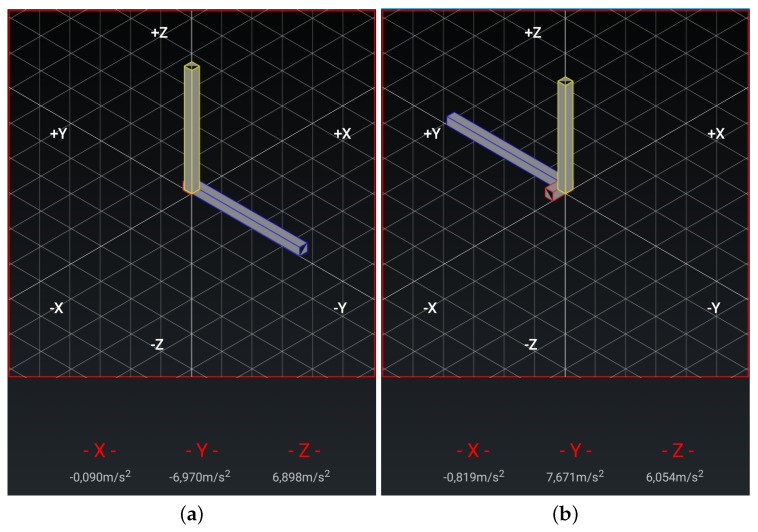
Gravity for the arm strength test: (**a**) Initial; (**b**) Final.

- **Chair Stand test** To carry out this test item, the mobile device has to be attached to the leg as depicted in Figure 3a. At the beginning of each repetition the acceleration in the direction of the *y*-axis is about 0 m/s${}^{2}$, that is, the user is sat and the phone is almost parallel to the floor. Then the user has to stand up placing the phone upside down and perpendicular to the floor, being the acceleration in the *y* axis about −9.8 m/s${}^{2}$. Finally the repetition is completed once the acceleration in *y* becomes 0 again. Figure 4 shows the values of the acceleration for this exercise when the user is standing and when is sit, while Figure 5 shows the evolution of the acceleration in the *y*-axis during several iterations. Note that the changes of the acceleration in the *z*-axis is not relevant in this case. This change is due to slightly changes in the phone height when attached to the leg and the acceleration in the *x* axis is not relevant and normally is close to zero. The datum point in the acceleration for the classification is the change in the value of the acceleration in the *y*-axis when it reaches −9.8 m/s${}^{2}$ indicating that the user is completely vertical. Finally, it is worth remarking that when designing our system, we considered more complex machine learning based classifiers. Nevertheless, these type of classifiers are not suitable to provide real time operation in a low resource smartphone. Therefore, giving that the performance of the proposed detection algorithm is good enough in practice, we decided to keep it to ensure the reliability of the system and the real time performance even with a low cost smartphone. However the inclusion of more sophisticated movement detection algorithms for powerful smartphones will be considered as future work.
- **Arm curl test** In this case the user has the arm fully extended with the fist towards down, and with the mobile device attached to the forearm, leaving it upside down as in Figure 3b. In this posture, the acceleration in *y* is negative. At the mid point of the exercise the acceleration on *y* becomes positive and higher to 7.5 m/s${}^{2}$, finally, the repetition is considered as completed when the acceleration comes back to the initial state. In Figure 6a,b we can observe both the acceleration at the beginning and at the midpoint of the exercise respectively.
- **2-min step in place test** To accomplish this test item the phone should be attached in the same way than for the chair stand test, (see Figure 3a). At the beginning the user is standing, and so the phone is upside down and perpendicular to the floor, being the acceleration in *y* around −9.8 m/s${}^{2}$. At the mid point of the exercise the acceleration in *y* evolves to 0 m/s${}^{2}$ (the user will be sit and so the phone will be almost parallel to the floor). The repetition is complete once the acceleration comes back to the initial state.
- **Chair sit and reach test and back scratch test** For this test item we have not implemented yet the feature that allows assessing the distance between the two hands, therefore in this first version of the platform this distance needs to be measured and introduce manually in the system by the practitioner. For future implementation, the option that we are considering is the inclusion of image analysis techniques to determine this distance in both tests [42] from a calibrated image taken during the test execution. We are aware that this is a valid option when the system is used by a health professional to monitor and track patients, but is difficult to use by an end-user, in that second case probably additional sensors will be required.
- **8-foot up and go test** This test item does not requires the patient to attach the phone, since the only sensor required is the chronometer. Therefore either the patient or the health practitioner has to start and stop the chronometer in the app, and the completion time will be automatically updated in the patient records.

#### 3.1.3. User Interface

**Figure 7:**
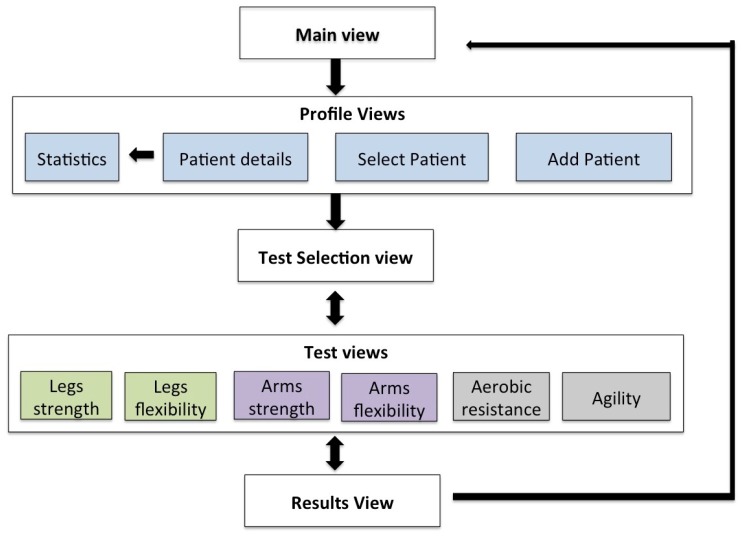
Mobile Senior Fitness Test (m-SFT) views flow.

**Figure 8:**
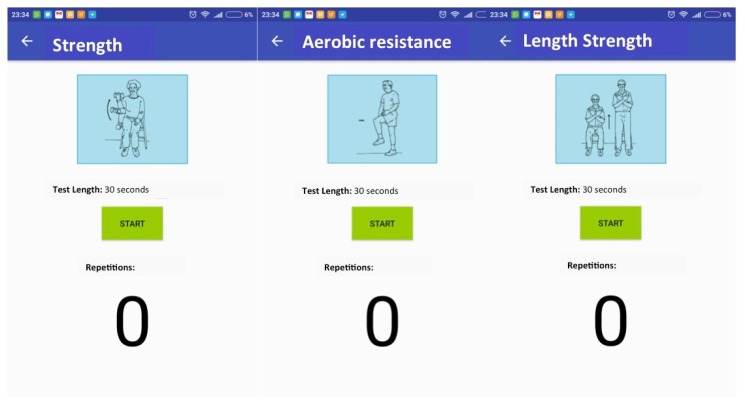
Tests views.

**Figure 9:**
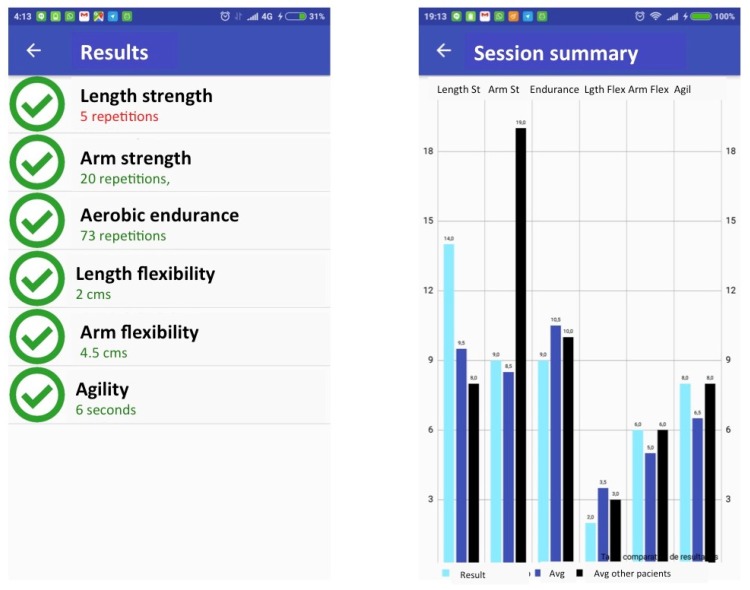
Results views.

In the following a detailed explanation of each of the views that compose the m-SFT’s UI is given. The navigation flow between them is presented in Figure 7 and these views are depicted in Figure 8 and Figure 9.

When the app is launched, the practitioner is directed to a view with a list of all the registered patients and the option of including a new one. To add a new patient to the system, the practitioner should provide the following patient’s information: ID, birth date, sex and, if desired, a picture of the patient. Alternatively, by selecting one of the patients in the main list, the practitioner would access the patient’s record that include the number of test sessions accomplished, the results and statistics, and the uncompleted sessions. Once the practitioner has chosen a patient, he/she can start a new test session. To do so the practitioner will be directed the test menu view, where the test item to do has to be selected. If the session has been resumed it will appear the completed test item among with the patient’s performance. Once the practitioner selects a test item, a view with the instructions appears, including pictures explaining how to attach the device and how to perform the test. After pressing the button continue the view of the specific test item is displayed showing its duration in seconds of the test (this parameter can be adjusted by the practitioner), the picture with the instructions for the test and the number of repetitions if required. Once the phone is properly attached to the patient and he/she is ready to go, the practitioner can press start and the device will start registering the number of repetitions and the time. Once the time is over they device rings and stops registering the repetitions.

To asses the overall patient’s performance in the whole Senior Fitness Test the system will evaluate whether the results are between the normal interval depending on the sex and the age of the patient according to the threshold pointed out in [14]. Notice that all the texts in the application are in Spanish, since the target practitioners and patients for the study were Spaniards. However the application can be easily translated to English.

### 3.2. m-SFT Evaluation

As mentioned the proposed system has been designed as a cost-effective and easy-to-use tool for a health practitioner to record and asses the physical condition of older adults. In this section, we present an preliminary analysis of the technical reliability and usability of the proposed system.

In order to carry out the technical reliability evaluation, seven volunteers, four females and three males ranging from 53 to 61 years old were assessed using the traditional test protocol described in Section 2.2 and using the m-SFT tool. In this first stage, we selected this population to obtain an approximation to the tool technical reliability. A higher sample with an adequate range of years will be needed to obtain solid conclusions; a future evaluation about the patient’s condition classification capacity will be performed. An experienced physiotherapist performed this evaluation. Before performing the assessment, the volunteers were informed of the research aims, risks, and benefits of participation. Next, they read and signed an informed consent form. The participants dressed comfortably and they were guided through the different tests. Participants were evaluated the same day at two different times, with a resting period of one hour [36]. In order to minimize the effect of measuring the same person in two different times, the assessment method was randomized using the coin toss method. The results obtained for each participant in both test modalities are shown in Table 2.

Before performing the statistical analysis, the results obtained were translated to a spreadsheet. Concerning these data, an analysis of the inter-rater reliability was performed (Table 3) using the SPSS version 24.0 for Mac (IBM Corporation, Armonk, NY, USA). The statistical analysis of the intraclass correlation coefficient (ICC) (ρ), Cronbach’s α estimator, and Bland-Altman plots were carried out and the agreement degree between the two assessment approaches is shown in Table 3. In order to interpret the results please note that an ICC (ρ) below 0.4 indicates poor inter-rater reliability; between 0.4 to 0.75 means fair to good reliability; and over 0.75 the reliability can bee considered as excellent [43,44]. In the case of the Cronbach’s α results below 0.5 were considered unacceptable; between 0.5 to 0.9 the acceptability ranges from poor to good and the values above 0.9 were considered as excellent [45].

**Figure 10:**
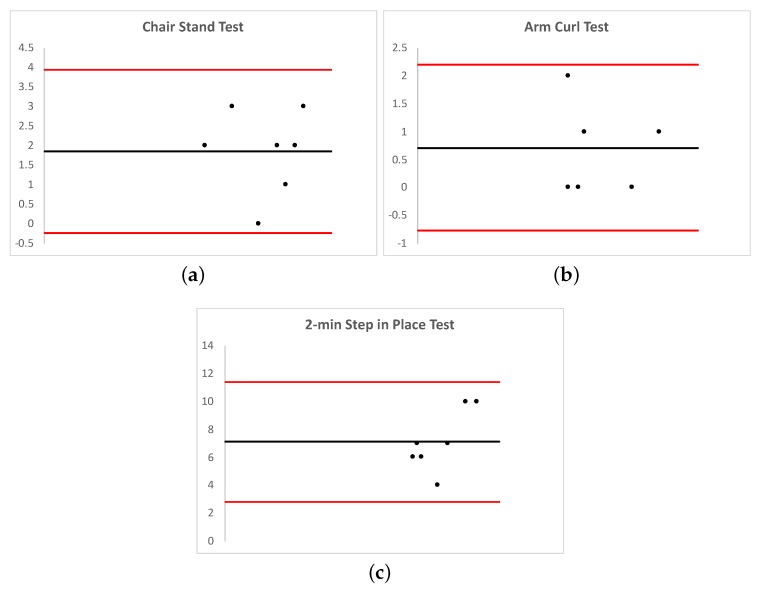
Agreement analysis between SFT and m-SFT assessment through Bland-Altman plots: (**a**) Chair Stand Test, (**b**) Arm Curl Test, and (**c**) 2-min Step in Place Test. The mean of differences ($\bar{x}$) is represented by a black line, while the limits of agreement ($\bar{x}\pm 1.96\sigma_{X}$) are depicted in red.

Furthermore, the graphical representation of the agreement between two techniques, known as Bland-Altman plots [46], was used to help understand the measurements of the two procedures against their averages. In that way, we plotted the results of those tests based on the smartphone sensors (Figure 10).

In views of these results, we can state that the proposed system has high technical reliability. All the measured variables provided high statistic values, ICC (ρ) and Cronbach’s α above 0.9. Therefore m-SFT can be considered as a reliable tool to assess the physical condition of older people.

**Figure 11:**
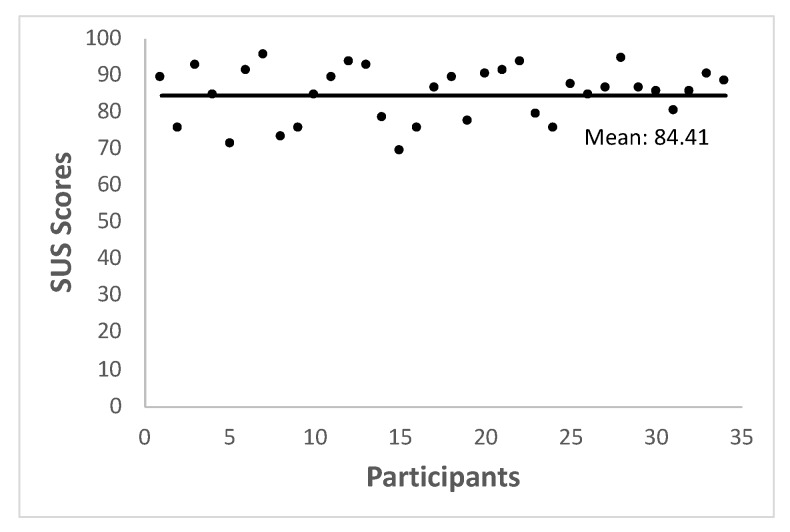
System Usability Scale (SUS) scores obtained from thirty four experts after using the proposed application.

Another important concern when developing an app for an specific segment of the population refers to its usability. Formally the usability can be considered as the facility of use of a tool or device giving to the user-device interaction a central role in the evaluation [6]. The usability of the proposed m-health app has been evaluated by employing the System Usability Scale (SUS) [47,48]. This scale, widely used in the industry, measures the user experience concerning various sort of technologies. The SUS consists on a ten items questionnaire answered by the tester using a five-point scale ranging from “strongly disagree” to “strongly agree”. The result is an estimated percentage of usability known as SUS score. Scores below 50% are considered as unacceptable whereas over 70% are considered as a good acceptability [49]. In our case, to carry out the usability evaluation, a total of 19 physiotherapists and 15 medical doctors (mean = 36.64 years; SD = 6.26 years; 18 males and 16 females) were asked to use the app during a small trial. First, a brief training on how to use the app were given to the experts, then, they were requested to carry out at least one whole SFT before answering to the SUS questionnaire. The average SUS score obtained is 84.4%, as shown in Figure 11, indicating high levels of acceptability, facility to use and confidence.

## 4. Discussion

In this contribution a new m-health system is described and evaluated, hypothesizing that the use of this type of technologies is useful for the clinical practice. In that way, the preliminary results suggest that a high agreement between the app and the traditional approach exists. Thus, it supposes a promising system to perform the analysis of the elderly physical condition, and also the follow-up of a specific intervention in an easy-to-use manner. Nonetheless, in the following paragraphs, some comments about the results need to be stated.

With regard to the technical reliability analysis the number of participants is not elevated, therefore establishing a strict conclusion based on this population is difficult. However, the ICC (ρ) and Cronbach’s α values are similar to previous validation studies. For example, the Knee Goniometer App [50], iHandy level app [51], or the timed-up-and-go test [52] also uses the inertial sensors and obtained ICC (ρ) and Cronbach’s α values above 0.90. Moreover, the analysis was performed in a laboratory controlled environment, and so some other issues may arise in a non-controlled clinical environment. With this regard, we should point out that the system has been tested only with healthy older adults able to perform bio-mechanically correct movements. However, in a real clinical environment, there might be some participants not able to execute a perfect movement, and consequently, in this case, the system may present some inaccuracies. These issues will be addressed in future versions of the platform.

With regard to the usability assessment, given that the SUS score obtained is over 80% m-SFT can be considered as an useful tool for the clinical environment. However, according to the experts’ opinion, although the current version and design are adequate to a laboratory test, it would be useful to add a more commercial-like appearance to the system in a future version. In that way, there are no usability analyses about an app employing SFT, but similar results were found in other apps. For example, TouchStream, an app to assess older adults with cancer, obtained a mean SUS of 78.7% [53]. Another app, assessing the fall risk in older adults obtained an average from 79 to 84 [54].

Given these promising results, some limitations have to be addressed. Formal validation of the m-SFT needs to be carried out, involving a larger number of senior participants and taking into consideration both laboratory and clinical conditions to assess the differences between them. The results obtained from the seven participants cannot be extrapolated to the general older adults population. Furthermore, although the tests present good reliability, they can change depending on the criteria for classification.

## 5. Conclusions

In this contribution we present a new intelligent m-health tool that asses the elderly physical condition by means of an electronic implementation of the well-known SFT. The proposed app is able to automatically evaluate the elderly physical condition by only using the built in sensors in an inexpensive Android Phone. The technical reliability of m-SFT has been tested by carrying out a comparative study between the original SFT and the proposed m-health system obtaining high agreement between both approaches (ICC between 0.93 and 0.99). Furthermore the system usability has been evaluated by independent health experts obtaining an average SUS score of 84.4, indicating high levels of acceptability, facility to use and confidence. Both results show that m-SFT is a reliable and easy-to-use m-health system for the evaluation of the elderly physical condition that also may be useful in intervention programs in order to electronically asses and record the patient’s evolution.

## Figures and Tables

**Table 1:** Senior Fitness Test (SFT) tests description.

Test	Measure	Description	60–69 Years	70–79 Year	80–89 Years
**Chair Stand Test (*n*)**	Strength of lower limbs	The number of times that person is able to stand up and sit without using the arms during a lapse of time of 30 s	14.0 (2.4)	12.9 (3.0)	11.9 (3.6)
**Arm Curl Test (*n*)**	Strength of upper limbs	The number of times that a person is able to fold the arm between 90 to 0 degrees holding a lift of 5 lb (2.27 kg) for women and 8 lb (3.63 kg) for men during a lapse of time of 30 s	19.8 (4.1)	18.2 (3.9)	16.5 (4.1)
**2-min Step in Place Test (*n*)**	Aerobic endurance	the number of times that starting in a stand up position, a person can raise the knees to a height halfway between the iliac crest and middle of the patella during the lapse of time of two minutes.	100.4 (9.0)	92.6 (16.0)	83.5 (22.6)
**Chair Sit and Reach Test (cm)**	Flexibility of the lower body	This test item asses the distance that a person can reach the toe (minus score) or beyond the toe (plus score) with fingers. The starting position is seated on the edge of a chair, with a leg extended straight in front of the hip with heel on floor flexed at 90°.	−1 (14)	−1 (15)	−8 (15)
**Back Scratch Test (cm)**	Flexibility of the upper limbs	Distance between (or the overlap of) the middle fingers behind the back when trying to touch the middle fingers of both hands together behind the back (measure to the nearest 1/2 inch).	3.0 (5.0)	−1 (8)	−5 (11)
**8-Feet (2.45 m) Up and Go Test (*s*)**	Agility and dynamic balance	The lapse of time a person takes to stand up from a chair, walk 8 feet (2.45 m) to and around a cone, and return to the chair (perform twice and measure time to the nearest 1/10th of a second, recording fastest time).	5.2 (0.6)	6.1 (1.2)	7.1 (2.0)

**Table 2:** Case study results.

Patient ID	1	2	3	4	5	6	7	8
**Gender**	Male	Female	Female	Male	Female	Female	Male	
**Age**	54	53	60	61	59	57	61	
**Chair Stand Test (SFT)**	14	15	12	14	10	16	12	
**Chair Stand Test (m-SFT)**	12	13	12	13	8	13	9	
**Arm Curl Test (SFT)**	23	16	15	20	14	16	15	
**Arm Curl Test (m-SFT)**	22	15	15	20	14	15	13	
**2-min Step in Place Test (SFT)**	75	80	93	97	72	74	85	
**2-min Step in Place Test (m-SFT)**	69	76	83	87	66	67	78	
**Chair Sit and Reach Test (SFT)**	−1	3	5	−3	2.5	2	−2	
**Chair Sit and Reach Test (m-SFT)**	−1	3.5	5	−2.5	3	2	−2	
**Back Scratch Test (SFT)**	−3.5	1	2	−2.5	2.5	−1	−4	
**Back Scratch Test (m-SFT)**	−3	1.5	2.5	−2	2.5	−1	−3.5	
**8-Foot Up and Go Test (SFT)**	5.3	6.2	3.5	4.1	6.1	3.3	6	
**8-Foot Up and Go Test (m-SFT)**	5	6.4	3.6	4.5	5.4	3.5	6.2	

**Table 3:** Inter-rater reliability between traditional Senior Fitness Test and m-SFT.

Variable	ICC (ρ) ${}^{*}$	CI 95% of ICC ${}^{+}$	Cronbach’s α
**Chair Stand Test**	0.93	0.58–0.99	0.93
**Arm Curl Test**	0.99	0.86–0.99	0.99
**2-min Step in Place Test**	0.98	0.83–0.99	0.98
**Chair Sit and Reach Test**	0.99	0.98–0.99	0.99
**Back Scratch Test**	0.99	0.99–1	0.99
**8-Foot Up and Go Test**	0.97	0.85–0.99	0.97

${}^{*}$ ICC (ρ) was calculated using a one-way random model. ${}^{+}$ ICC indicates the intra-class correlation coefficient. CI, confidence interval.
